# COL3A1 and MMP9 Serve as Potential Diagnostic Biomarkers of Osteoarthritis and Are Associated With Immune Cell Infiltration

**DOI:** 10.3389/fgene.2021.721258

**Published:** 2021-08-27

**Authors:** Shushan Li, Haitao Wang, Yi Zhang, Renqiu Qiao, Peige Xia, Zhiheng Kong, Hongbo Zhao, Li Yin

**Affiliations:** Department of Orthopedic Surgery, The First Affiliated Hospital of Zhengzhou University, Zhengzhou, China

**Keywords:** osteoarthritis, immune cell infiltration, bioinformatics, GEO, diagnostic markers

## Abstract

**Background:**

Osteoarthritis (OA) is one of the most common age-related degenerative diseases. In recent years, some studies have shown that pathological changes in the synovial membrane occur earlier than those in the cartilage in OA. However, the molecular mechanism of synovitis in the pathological process of OA has not been elucidated. This study aimed to identify novel biomarkers associated with OA and to emphasize the role of immune cells in the pathogenesis of OA.

**Methods:**

Microarray datasets were obtained from the Gene Expression Omnibus (GEO) and ArrayExpress databases and were then analyzed using R software. To determine differential immune cell subtype infiltration, the CIBERSORT deconvolution algorithm was used. Quantitative reverse transcription PCR (qRT-PCR) was used to determine the relative expressions of selected genes. Besides, Western blotting was used to assess the protein expression levels in osteoarthritic chondrocytes.

**Results:**

After analyzing the database profiles, two potential biomarkers, collagen type 3 alpha 1 chain (*COL3A1*), and matrix metalloproteinase 9 (*MMP9*), associated with OA were discovered, which were confirmed by qRT-PCR and Western blotting. Specifically, the results revealed that, as the concentration of IL-1β increased, so did the gene and protein expression levels of *COL3A1* and *MMP9*.

**Conclusion:**

The findings provide valuable information and direction for future research into novel targets for OA immunotherapy and diagnosis and aids in the discovery of the underlying biological mechanisms of OA pathogenesis.

## Introduction

Osteoarthritis (OA), one of the most common age-related degenerative diseases, is characterized by osteophyte formation, cartilage degeneration, and synovial inflammation (Luo et al., 2018; Wang et al., 2018), which eventually lead to loss of joint function due to the limited repair capacity of the cartilage (Kim et al., 2018). However, the pathology of OA is not fully understood, and there is no treatment available to prevent or slow its progression (Wang et al., 2019). As a result, early diagnosis and treatment are preferred to improve joint function and alleviate joint pain.

According to recent research, the degenerative changes in the synovial membrane in OA occur earlier than those in the cartilage (Sakurai et al., 2019). OA synovitis is most likely caused by an innate immune response and is mediated by the expression of matrix-degrading enzymes, inflammatory cytokines, and chemokines (Gómez et al., 2015; Qadri et al., 2020). In several studies, the degree of synovitis has been validated as a strong predictor of OA, particularly in its early stages (Conaghan et al., 2010; Mathiessen and Conaghan, 2017). Immune responses are widely acknowledged to play an important role in the pathogenesis of OA (Daheshia and Yao, 2008; Han et al., 2018; Jenei-Lanzl et al., 2019). Pro-inflammatory cytokines promote chondrocyte apoptosis and cartilage matrix proteolysis (Utomo et al., 2016; Mobasheri et al., 2017). Furthermore, inflammatory suppression may aid in alleviating cartilage degradation in OA (Kapoor et al., 2011). However, the molecular mechanism of synovitis in the pathological process of OA has not been elucidated.

In the present study, microarray data from synovial membrane and cartilage samples in aged OA patients were integrated and the diagnostic biomarkers of OA were determined. The CIBERSORT algorithm method was then used to analyze immune cell infiltration in “normal” synovial membrane and OA synovial membrane. Furthermore, osteoarthritic chondrocytes (OA-CH) were stimulated with interleukin 1β (IL-1β) to establish a standardized *in vitro* OA model; the relationship between IL-1β and diagnostic biomarkers [collagen type 3 alpha 1 chain (*COL3A1*) and matrix metalloproteinase 9 (*MMP9*)] was determined by quantitative reverse transcription PCR (qRT-PCR) and Western blotting. This study aimed to identify novel biomarkers associated with OA and to emphasize the importance of immune cells in the pathogenesis of OA. The findings of the current study could lead to new OA diagnostic targets.

## Materials and Methods

### Identification of Differentially Expressed Genes

**FIGURE 1:**
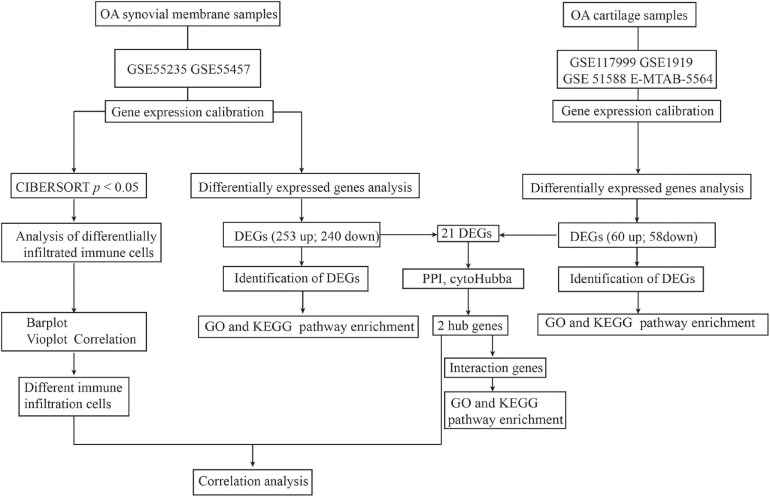
Workflow of the entire study.

Figure 1 depicts the study workflow. Microarray datasets of synovial membrane (GSE55235 and GSE55457) and cartilage (GSE117999, GSE1919, GSE51588, and E-MTAB-5564) samples were obtained from the Gene Expression Omnibus (GEO)^1^ and ArrayExpress^2^ databases. The ComBat function in the sva R package^3^ was used to correct inter-batch differences in the different datasets. The limma package^4^ in R was used to normalize and screen differentially expressed genes (DEGs) by comparing the expression levels in the synovial membrane from normal joints to those from OA joints. DEGs with | logFC| > 2 and an adjusted *p*-value < 0.05 were considered significantly expressed.

### GO and KEGG Pathway Enrichment

The cluster Profiler^5^ in R package was used to perform Gene Ontology (GO) annotation and Kyoto Encyclopedia of Genes and Genomes (KEGG) pathway enrichment analysis of the enriched DEGs. A value of *p* < 0.05 was considered statistically significant.

### Construction of a PPI Network and Analysis of Hub Genes

A protein–protein interaction (PPI) network was established using STRING,^6^ an online PPI establishment tool. The genes with a combined score of 0.4 were selected and used to establish the PPI network. Furthermore, the Cytohub plugin in Cytoscape version 3.8.0^7^ was used to identify hub genes using the degree method (degree > 4).

### CIBERSORT Analysis of Immune Cell Infiltration

The CIBERSORT deconvolution algorithm^8^ was used to determine differential immune cell subtype infiltration between normal and OA synovial membrane samples. The difference in immune cell density between the normal and rheumatoid arthritis (RA) groups was visualized using a heatmap package in R version 3.6.0. The Wilcoxon signed-rank test was used to determine the statistical significance of the differences in immune cell infiltration between the two groups as depicted by violin plots.

### Ethical Statement

The use of human material was approved by the local ethics committee of The First Affiliated Hospital of Zhengzhou University (reference no. 2021-KY-0338-002), and all patients provided written consent.

### IL-1β Stimulation of OA Chondrocytes

Osteoarthritic chondrocytes (2 × 10^5^, passages 2–4) were cultured in six-well plates with DMEM F12 medium [supplemented with 10% normal fetal calf serum (FCS) and 1% penicillin–streptomycin], stimulated with IL-1β (1, 5, and 10 ng/ml) (MAN0004230; Thermo Fisher Scientific, Waltham, MA, United States), and harvested for RNA and protein isolation after 24 and 48 h, respectively.

### RNA Extraction and Real-Time PCR Analysis

Total RNA was isolated from the cells using the Absolutely RNA Miniprep Kit (Agilent Technologies, Santa Clara, CA, United States) according to the manufacturer’s instructions and reverse-transcribed into complementary DNA (cDNA) using the AffinityScript QPCR cDNA Synthesis Kit (#600559; Agilent Technologies, Santa Clara, CA, United States). Subsequently, an MX3005P QPCR System (Agilent Technologies, Santa Clara, CA, United States) was used to perform real-time PCR for messenger RNA (mRNA) expression with Brilliant III Ultra-Fast SYBR^®^ Green QPCR Master Mix (#600882; Agilent Technologies, Santa Clara, CA, United States). The primer sequences of the target genes were as follows: *MMP9* (Fwd: 5′-GTA CCA CGG CCA ACT ACG AC-3′; Rev: 5′-GCC TTG GAA GAT GAA TGG AA-3′), *COL3A1* (Fwd: 5′-CTTCTCTCCAGCCGAGCTTC-3′; Rev: 5′-TGTGTTTCGTGCAACCATCC-3′), *TBP* (Fwd: 5′-TTGTAC CGCAGCTGCAAA AT-3′; Rev: 5′-TATATTC GGCGTTTCGGGCA-3′), and *GAPDH* (Fwd: 5′-CT GACTTCAACAGCGACACC-3′; Rev: 5′-CC CTGTTGCTGTAGCCAAAT-3′). All genes were analyzed relatively, calibrated to the expression of the control cell culture groups, and normalized to *GAPDH* and *TBP*.

### Protein Extraction and Western Blotting Analysis

Osteoarthritic chondrocytes were washed twice with cold phosphate-buffered saline (PBS) and lysed with RIPA buffer (Thermo Fisher Scientific, Waltham, MA, United States) containing proteinase inhibitors (Roche, Basel, Switzerland). The concentration of cellular protein was determined using a BCA protein kit assay. Cell lysates were mixed with sodium dodecyl sulfate (SDS) sample loading buffer (#B7053; Sigma-Aldrich, Taufkirchen, Germany), boiled for 5 min at 95°C, and then subjected to 10% SDS-PAGE. After electrophoretic separation, the proteins were transferred to 0.22-mm polyvinylidene fluoride (PVDF) membranes (Roche, Penzberg, Germany). Blot membranes were blocked with 5% bovine serum albumin (BSA) for 1 h at room temperature and incubated with primary antibodies on a shaker overnight at 4°C. The membranes were then washed and incubated with the appropriate horseradish peroxidase-coupled secondary antibodies (Santa Cruz Biotechnology and Jackson ImmunoResearch, West Grove, PA, United States). The proteins were examined using enhanced chemiluminescence (ECL) detection reagents (Thermo Scientific, Waltham, MA, United States) and signals were normalized to β-actin. The following primary antibodies were used in this study: COL3A1 (1:1,000, #ab838292; Abcam, Cambridge, MA, United States), MMP9 (1:200, #sc-393859; Santa Cruz, Heidelberg, Germany), and β-actin (1:5,000, #ab8227; Abcam, Cambridge, MA, United States).

### Statistical Analysis

R version 3.6.0 was used to perform bioinformatics analyses, and a *p*-value < 0.05 was considered statistically significant. Correlations were determined using Pearson’s correlation coefficient, with | *R*| < 0.5 indicating a weak correlation. For qRT-PCR and Western blotting analyses, an unpaired Student’s *t*-test was used for two groups and one-way ANOVA was used for groups of more than two. Each assay was replicated and repeated in at least three independent experiments. A value of *p* < 0.05 was considered statistically significant.

## Results

### Identification of DEGs

Microarray datasets of synovial membrane (GSE55235 and GSE55457) and cartilage samples (GSE117999, GSE1919, GSE51588, and E-MTAB-5564) were obtained from the GEO and ArrayExpress databases. Before analyzing the DEGs, raw data were preprocessed for batch correction and normalization. Gene expression levels with | logFC| > 1 and an adjusted *p*-value < 0.05 were considered differentially expressed. As a result, 253 upregulated and 240 downregulated DEGs were identified in synovial membrane samples when compared to normal samples, while 60 upregulated and 58 downregulated DEGs were identified in cartilage samples, as shown in Figure 1.

### Function Annotation of DEGs

**FIGURE 2:**
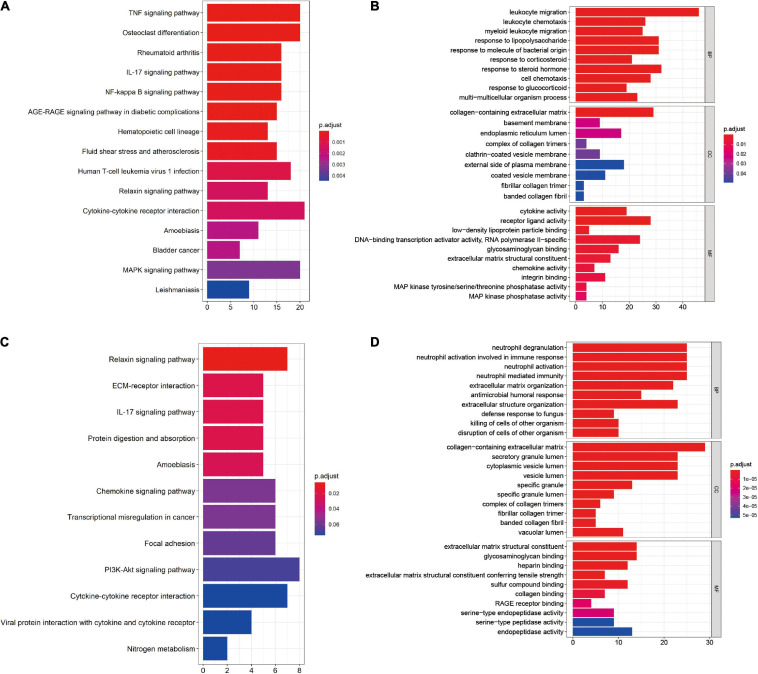
Functional enrichment of the differentially expressed genes (DEGs) in the synovial membrane and cartilage samples. **(A,B)** Kyoto Encyclopedia of Genes and Genomes (KEGG) pathway enrichment and Gene Ontology (GO) analysis of the DEGs in synovial membrane samples. **(C,D)** KEGG pathway enrichment and GO analysis of the DEGs in cartilage samples.

Kyoto Encyclopedia of Genes and Genomes pathway enrichment and GO functional enrichment of DEGs were performed to investigate the mechanisms involved in the pathogenesis of OA. KEGG pathway enrichment revealed that synovial membrane DEGs were mainly enriched in cytokine–cytokine receptor interaction, mitogen-activated protein kinase (MAPK) pathway, and tumor necrosis factor (TNF) pathway (Figure 2A), while DEGs from cartilage samples were enriched in the PI3K/AKT pathway, cytokine–cytokine receptor interaction, and the chemokine pathway (Figure 2C). Furthermore, GO functional enrichment analysis revealed that synovial membrane DEGs were mainly involved in leukocyte migration, regulation of inflammatory response, and collagen-containing extracellular matrix (Figure 2B), while cartilage DEGs were mainly involved in the collagen-containing extracellular matrix, neutrophil degranulation, and neutrophil activation involved in immune response (Figure 2D). These findings suggest that DEGs in both the synovial membrane and cartilage are involved in immune response signaling pathways and that the immune system plays a critical role in the pathological processes of OA.

### Screening and Validation of *MMP9* and *COL3A1* Hub Genes

**FIGURE 3:**
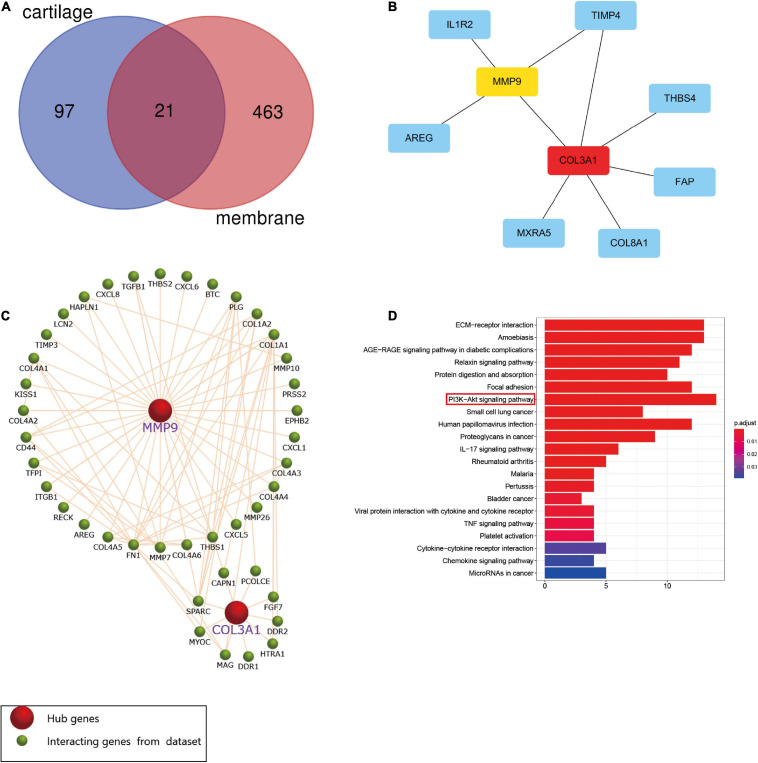
Screening of hub genes and functional analysis. **(A)** Twenty-one differentially expressed genes (DEGs) intersected between the cartilage samples and the synovial membrane samples. **(B)** Protein–protein interaction (PPI) network of the 21 DEGs and two hub genes screened by the degree method (degree > 4) using cytoHubba. A higher ranking is represented by a redder color. **(C)** *MMP9* and *COL3A1* interacting genes indicated using Funrich software. **(D)** Kyoto Encyclopedia of Genes and Genomes (KEGG) pathway enrichment analysis of *MMP9* and *COL3A1* interacting genes.

The Venn diagram showed that 21 DEGs from the synovial membrane and cartilage samples overlapped (Figure 3A). The PPI network between the overlapping DEGs was constructed and two hub genes, *MMP9* and *COL3A1*, were filtered out (Figure 3B) by the degree method (degree > 4) using cytoHubba. The Funrich software was used to display the 41 interacting genes to better understand the functions of *MMP9* and *COL3A1* (Figure 3C). In addition, KEGG enrichment revealed that 41 *MMP9* and *COL3A1* interacting genes were involved in the PI3K/AKT pathway, IL-17 pathway, TNF pathway, and other immune-related pathways (Figure 3D and Table 1). These findings imply that MMP9 and COL3A1 are involved in the pathophysiological inflammatory processes that lead to OA.

**TABLE 1:** Signaling pathway enrichment of *MMP9* and *COL3A1* interacting genes.

ID	Description	*p*-value	*p*_adjust_	Count
hsa04151	PI3K–Akt signaling pathway	4.70E–11	6.11E–10	14
hsa04512	ECM–receptor interaction	9.39E–18	8.55E–16	13
hsa05146	Amoebiasis	7.07E–17	3.22E–15	13
hsa04933	AGE–RAGE signaling pathway in diabetic complications	2.89E–15	8.77E–14	12
hsa04510	Focal adhesion	1.38E–11	2.09E–10	12
hsa05165	Human papillomavirus infection	4.48E–09	4.53E–08	12
hsa04926	Relaxin signaling pathway	2.35E–12	5.35E–11	11
hsa04974	Protein digestion and absorption	7.43E–12	1.35E–10	10
hsa05205	Proteoglycans in cancer	1.11E–07	1.01E–06	9
hsa05222	Small cell lung cancer	2.97E–09	3.38E–08	8
hsa04657	IL–17 signaling pathway	2.19E–06	1.81E–05	6
hsa05323	Rheumatoid arthritis	3.88E–05	0.000295	5
hsa04060	Cytokine–cytokine receptor interaction	0.007225	0.034604	5
hsa05206	MicroRNAs in cancer	0.008991	0.03896	5
hsa05144	Malaria	5.26E–05	0.000368	4
hsa05133	Pertussis	0.000271	0.001763	4
hsa04061	Viral protein interaction with cytokine and cytokine receptor	0.000772	0.004389	4
hsa04668	TNF signaling pathway	0.00118	0.006314	4
hsa04611	Platelet activation	0.001719	0.008692	4
hsa04062	Chemokine signaling pathway	0.008221	0.037406	4
hsa05219	Bladder cancer	0.000652	0.003953	3

### Analysis of Immune Cell Infiltration in Normal and OA Synovial Membrane Samples

**FIGURE 4:**
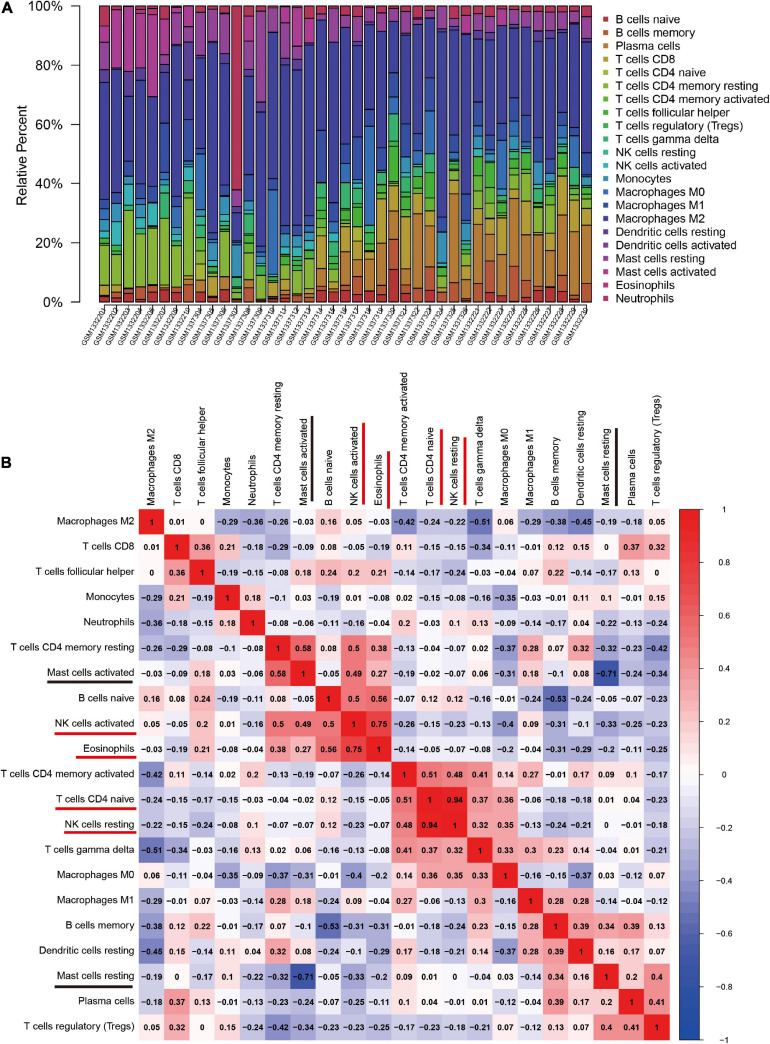
The landscape and correlation heatmap of immune infiltration in synovial membrane samples between the normal and osteoarthritis (OA) groups. **(A)** Relative distribution of 22 immune cells in all samples. **(B)** Correlation heatmap of immune cells in all samples. Red squares indicate positive correlation and blue squares indicate negative correlation; the deeper colored squares indicate stronger correlations.

The CIBERSORT algorithm was, for the first time, used to reveal the landscape of the differentially infiltrated immune cells in “*normal*” *versus* OA synovial membrane samples in 22 subpopulations of immune cells. The heatmap shows the proportion of immune cells in the two groups (Figure 4A).

The correlation heatmap of the 22 immune cell subtypes showed that two pairs of immune cells [active natural killer (NK) cells and eosinophils, and naive CD4 T cells and resting NK cells] were positively correlated and that two immune cell subtypes (activated mast cells and resting mast cells) were negatively correlated (Figure 4B).

**FIGURE 5:**
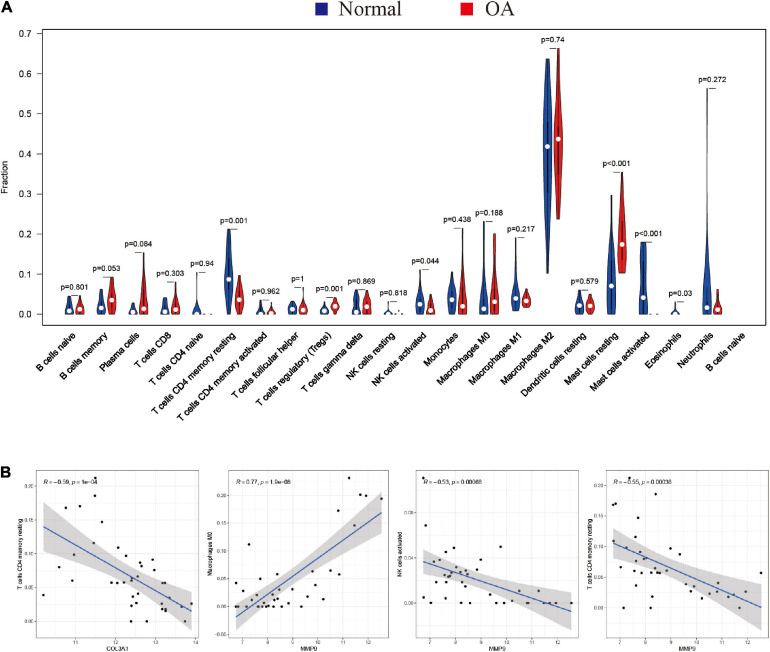
Characterization of immune cell infiltration in normal and osteoarthritis (OA) samples and the correlation between hub gene expression and immune cell infiltration. **(A)** Violin plot showing the differentially infiltrated immune cells of a proportion of the 22 immune cell types. The red underline shows significant difference in the immune cell infiltration between the normal and rheumatoid arthritis (RA) groups. A value of *p* < 0.05 was considered to be statistically significant. **(B)** Correlation coefficient (*R*) > 0.5; *p* < 0.05 was considered statistically significant.

**FIGURE 6:**
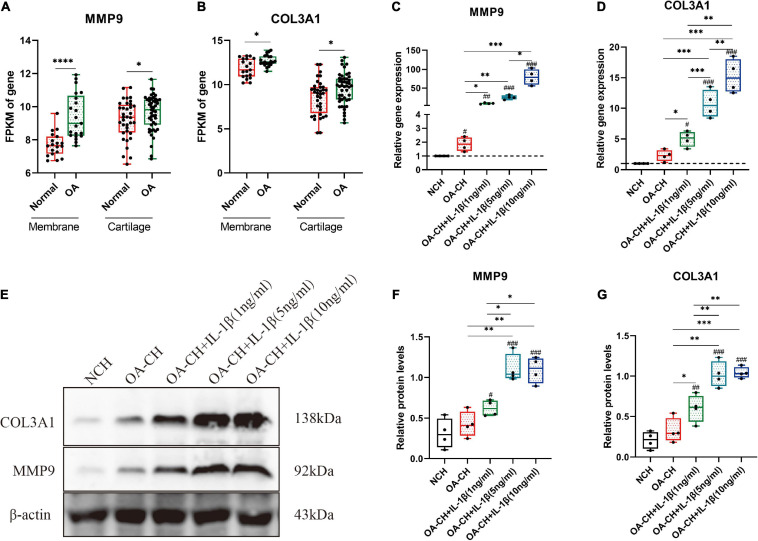
Validation of the hub genes (*COL3A1* and *MMP9*) by quantitative reverse transcription PCR (qRT-PCR) and Western blotting. **(A,B)** Fragments per kilobase of exon model per million mapped fragments (FPKM) of *COL3A1* and *MMP9* in synovial membrane and cartilage samples. *p < 0.05; ****p < 0.001, unpaired Student’s *t*-test. **(C,D)** Gene expression levels of *COL3A1* and *MMP9* in non-osteoarthritic chondrocytes (NCH) and osteoarthritic chondrocytes (OA-CH) treated with different concentrations of IL-1β. **(E–G)** Western blotting was used to determine the protein expression levels of *COL3A1* and *MMP9* in NCH and OA-CH treated with different concentrations of IL-1β. Significant difference to control (NCH): ^#^*p* < 0.05; ^##^*p* < 0.01; ^###^*p* < 0.001. *Significant difference between groups: *p < 0.05; **p < 0.01; ***p < 0.001. One-way ANOVA with Newman–Keuls multiple comparison test. All values represent the mean ± standard deviation (*n* = 4).

Furthermore, the violin plot of the differentially infiltrated immune cells showed that regulatory T cells (Tregs) and resting mast cells had the highest infiltration rates in OA samples compared with “normal” samples, whereas resting CD4^+^ memory T cells, activated NK cells, activated mast cells, and eosinophils were less prominent in OA samples (Figures 5A, 6B).

### Correlation Between Hub Genes (*MMP9* and *COL3A1*) and Immune Cell Infiltration

Spearman’s correlation analysis was performed to determine the association between the hub genes (*MMP9* and *COL3A1*) and the infiltrated immune cell subtypes in the synovial membranes of both groups (Figure 5B). *MMP9* and *COL3A1* were found to be negatively correlated with resting CD4 memory T cells, whereas *MMP9* was found to be positively correlated with M0 macrophages and negatively correlated with activated NK cells.

### Validation of Hub Genes (*COL3A1* and *MMP9*) by qRT-PCR and Western Blotting

The fragments per kilobase of exon model per million mapped fragments (FPKM) values of *COL3A1* and *MMP9* were significantly higher in the OA cartilage and synovial membrane compared with those in normal samples (Figures 6A,B). To validate the expressions of *COL3A1* and *MMP9* in chondrocytes, qRT-PCR and Western blotting were used to determine the gene and protein expressions in non-osteoarthritic chondrocytes (NCH), OA–CH, and OA–CH treated with different concentrations of IL-1β. As shown in Figures 6C,D, the gene expression levels of both *COL3A1* and *MMP9* increased in the OA-CH group, and IL-1β promoted the expressions of *COL3A1* and *MMP9*. Notably, the expressions of *COL3A1* and *MMP9* increased as the concentration of IL-1β increased. Furthermore, in the presence of IL-1β, the protein levels of *COL3A1* and *MMP9* increased (Figures 6E–G). These findings suggest that the gene and protein expression levels of *COL3A1* and *MMP9* were positively correlated with the degree of inflammation and the inflammatory activity.

## Discussion

Osteoarthritic is a type of chronic joint disease that is characterized by cartilage degeneration, hyperosteogenia, and synovitis (Xie and Chen, 2019). Accumulating evidence suggests that pro-inflammatory cytokines, such as IL-1β, TNF, and IL-6, play a role in the pathophysiology of OA (Robinson et al., 2016; Urban and Little, 2018). Previous research has focused on the molecular mechanism of OA in the cartilage or chondrocytes while ignoring the synovial membrane. In recent years, an increasing number of studies have shown that synovitis plays a critical role in the pathological process of OA, from the early to the end stages (Atukorala et al., 2016; Huang et al., 2018; Griffin and Scanzello, 2019). Additional research has revealed changes in immune cell infiltration in OA synovial membrane samples (Moradi et al., 2014; Penatti et al., 2017; Rosshirt et al., 2019). However, no study has been conducted to investigate the inflammatory relationship between the synovial membrane and cartilage. In the present study, the gene expression profiles of the synovial membrane and cartilage were combined to identify the important hub genes associated with synovitis in OA.

Differentially expressed genes in the synovial membrane and cartilage were separately analyzed; GO annotation and KEGG pathway enrichment were used to reveal the functions of these DEGs. Our results also showed that both synovial membrane and cartilage DEGs were mainly involved in inflammatory pathways and pathological processes, which was consistent with previous studies (Qin et al., 2012; Hou et al., 2013; Chen et al., 2018). Furthermore, the immune response occurred in the synovial membrane and cartilage, indicating that synovitis plays a critical role in the pathological process of OA.

The hub genes *COL3A1* and *MMP9* were identified and their function validated using Funrich software and by KEGG pathway enrichment, respectively. Besides, *COL3A1* and *MMP9* interacting genes were found to be mainly involved in the PI3K/AKT signaling pathway, extracellular matrix (ECM) receptor interaction, and other inflammatory signaling pathways (IL-17 signaling pathway, cytokine–cytokine receptor interaction, TNF signaling pathway, and chemokine signaling pathway). Numerous studies have reported that inflammatory signaling pathways, including the PI3K/AKT, IL-17, TNF, NF-κB, and MAPK signaling pathways, are involved in the osteoarthritic process (Balabko et al., 2015; Zhang et al., 2018; Han et al., 2019; Li and Zheng, 2019). These findings suggest that *COL3A1* and *MMP9* play important roles in the inflammatory signaling pathways linked to OA.

To further investigate the effect of immune cell infiltration in OA, CIBERSORT was used to perform a comprehensive analysis of OA immune infiltration. The results showed increased infiltration of Tregs and resting mast cells, which contributed to the occurrence and development of OA. Moradi et al. (2014) found that Tregs are enriched in the synovial membrane of OA patients and correlated with the levels of inflammatory factors (IL-10 and TGF-β) (Xia et al., 2017). Resting mast cells were found in high numbers in OA synovial tissue, which is associated with structural damage in OA patients (de Lange-Brokaar et al., 2016). These findings and other related research indicate that Tregs and resting mast cells play an important role in OA. In this study, the relationship between the immune cell subtypes in OA was investigated; the results showed that two pairs of immune cells (activated NK cells and eosinophils, and naive CD4 T cells and resting NK cells) were positively correlated and that two immune cell subtypes (activated and resting mast cells) were negatively correlated. However, the correlation between the immune cell subtypes requires further experimental validation.

The relationship between hub gene expression and immune cell infiltration was also analyzed. The results showed that the expressions of both *MMP9* and *COL3A1* were negatively correlated with resting CD4 memory T cells, while the expression of *MMP9* was positively correlated with M0 macrophages and negatively correlated with activated NK cells. We hypothesized that *MMP9* and *COL3A1* inhibited the immune response by reducing the resting CD4 memory T cells and activated NK cells and that *MMP9* increased M0 macrophages to induce inflammation in the course of OA. However, further research is needed to validate these assumptions on the relationship between hub genes and immune cells.

To investigate the correlation between the hub genes (*MMP9* and *COL3A1*) and OA, qRT-PCR, and Western blotting were used to determine the gene and protein expression levels in chondrocytes. The results indicated that the gene expression levels of *MMP9* and *COL3A1* increased in OA–CH compared with those in NCH. Notably, the gene and protein expression levels of *COL3A1* and *MMP9* increased with an increase in IL-1β concentration. Evidence suggests that the expression levels of *COL3A1* increased in the early stages of OA and decreased in the later stages (Rai et al., 2019). Tang et al. (2018) also discovered that IL-1 increased the protein levels of *COL3A1* in synoviocytes. MMP9, also known as gelatinase B, is an enzyme that degrades the ECM components such as collagen, fibronectin, and laminin. MMP9 was found to be upregulated at the mRNA and protein levels in the cartilage and synovial membrane, as well as in the synovial fluid, and was found to be related to the severity of OA (Bollmann et al., 2021). These findings suggest that COL3A1 and MMP9 could be used as OA diagnostic biomarkers. However, more research is needed to determine the roles of COL3A1 and MMP9 in the progression of OA.

## Conclusion

In conclusion, the present study identified two potential OA biomarkers, *COL3A1*, and *MMP9*, which were confirmed by qRT-PCR and Western blotting analysis. Notably, the gene and protein expression levels of *COL3A1* and *MMP9* increased with an increase in IL-1β concentration. These findings provide valuable information and direction for future research into novel targets for OA immunotherapy and diagnosis and aid in the discovery of the underlying biological mechanisms of OA pathogenesis.

## Data Availability Statement

The original contributions presented in the study are included in the article/supplementary material, further inquiries can be directed to the corresponding author.

## Ethics Statement

The use of human material has been approved by the local ethics committee (reference number: 2021-KY-0338-002, The First Affiliated Hospital of Zhengzhou University) and the written consent of all patients has been obtained.

## Author Contributions

SL contributed to sample collection, experiment design, investigation, data curation, writing the original draft, and review and editing. HW and YZ helped with conceptualization and review and editing. RQ, PX, HZ, and ZK helped with the methodology, establishment of qRT-PCR, and bioinformatics analysis. LY made contributions to the conceptualization, review and editing, project administration, and funding acquisition. All authors proofread the final version of the manuscript.

## Conflict of Interest

The authors declare that the research was conducted in the absence of any commercial or financial relationships that could be construed as a potential conflict of interest.

## Publisher’s Note

All claims expressed in this article are solely those of the authors and do not necessarily represent those of their affiliated organizations, or those of the publisher, the editors and the reviewers. Any product that may be evaluated in this article, or claim that may be made by its manufacturer, is not guaranteed or endorsed by the publisher.

## Footnotes

1
http://www.ncbi.nlm.nih.gov/geo/

2
https://www.ebi.ac.uk/arrayexpress/

3
https://bioconductor.org/packages/release/bioc/html/sva.html

4
http://bioconductor.org/packages/release/bioc/html/limma.html

5
https://www.bioconductor.org/help/search/index.html?q=clusterProfiler/

6
http://string-db.org

7
https://cytoscape.org/

8
https://cibersort.stanford.edu/
